# Diversity-in-a-dish: A practical framework for hiPSC model development

**DOI:** 10.1016/j.stemcr.2026.102858

**Published:** 2026-03-19

**Authors:** Jesse Weidema, Hanna Lammertse, Martine de Vries, Christine Mummery, Megan Munsie, Nienke de Graeff

**Affiliations:** 1 Department of Medical Ethics and Health Law, Leiden University Medical Center, Leiden, the Netherlands; 2 Department of Anatomy and Embryology, Leiden University Medical Center, Leiden, the Netherlands; 3 hDMT, Institute for Human Organ and Disease Model Technologies, Leiden, the Netherlands; 4 Stem Cell Medicine, Murdoch Children’s Research Institute, Parkville, VIC, Australia; 5 Melbourne Medical School, University of Melbourne, Parkville, VIC, Australia

**Keywords:** human induced pluripotent stem cells, organ-on-chip, organoid, preclinical research, diversity, model validation

## Abstract

Human induced pluripotent stem cell (hiPSC) models inherit genetic and phenotypic variation from their donors that can influence differentiation, function, and disease phenotypes, with implications for model validity and generalizability. While diversity is widely discussed in biomedical research and extensively theorized in fields like genomics, comparable guidance remains limited in hiPSC research. Effectively, diversity-related decisions are often made implicitly without clear criteria for when inclusion is methodologically warranted. We address this by (1) analyzing how diversity is defined and operationalized in (pre)clinical research, (2) extending genomics frameworks to outline stem cell-specific recommendations for describing and reporting diversity, and (3) introducing a decision framework based on four criteria (experimental purpose, biological plausibility, platform readiness, and statistical power) to determine when and how diversity should be incorporated. The overall goal is to establish a shared basis for transparent and reproducible diversity-related design and reporting.

## Teaser

hiPSC models inherit donor variation that can shape experimental outcomes, yet decisions about when diversity should be included are often implicit. This perspective proposes a practical framework to make diversity-related design choices explicit, comparable, and methodologically justified across stem-cell-based preclinical research contexts.

## Introduction

Human induced pluripotent stem cells (hiPSCs) are transforming biomedical research by providing renewable sources of human cells for disease modeling, drug testing, and regenerative therapies (Rowe and Daley, 2019). Microphysiological systems (MPSs), including organoids and organ-on-chips (OoCs), extend this potential by reconstructing key structural and functional features of human tissues under controlled conditions (Low et al., 2021). Despite these advances, hiPSC repositories remain demographically skewed: comparative analyses of major collections show that large international repositories such as the California Institute for Regenerative Medicine (CIRM) and the Human Induced Pluripotent Stem Cells Initiative (HipSci) are dominated by lines of European ancestry, while national repositories in Asia, including the RIKEN Bioresource Research Center (BRC), the Center for iPSC Research and Application (CiRA), and the National Stem Cell Bank of Korea, primarily represent domestic populations, effectively leaving global variation unevenly captured (Ghosh et al., 2022).

While the exact role of ancestry in shaping hiPSC phenotypes remains largely unclear (Ghosh et al., 2022), a growing set of multi-donor studies demonstrates that both common and rare genomic variants can drive measurable differences in cellular function and disease-relevant traits. For instance, genomic background affects transcriptional responses to environmental exposures (Lin et al., 2025), immune responses and neurodegeneration risk (Andzelm et al., 2025), viral susceptibility (Mitchell et al., 2020; cf; Wells et al., 2023), and cellular morphology and differentiation capacity (Kilpinen et al., 2017; Tegtmeyer et al., 2024). Without broader donor representation, variant effects relevant to disease susceptibility, drug metabolism, and treatment response in underrepresented populations may remain undetected, which can in turn lead to increased clinical misdiagnoses and exacerbated health care disparities (Sirugo et al., 2019).

Initiatives addressing these gaps are now unfolding worldwide, including national projects in the United Kingdom (HipSci), the United States (All of Us), China (China 100,000 Genomes Project), Greece (GoGreece), Turkey (Turkish Genome Project), Saudi Arabia (Saudi Human Genome Program), and Brazil (Brazilian Initiative on Precision Medicine), as well as continent-wide projects across Africa (H3Africa) and Asia (GenomeAsia100K) (Ghosh et al., 2022; Hurrell et al., 2024; Nunes et al., 2025; Wall et al., 2019). Researchers have also developed practical methods to address underrepresentation, including workflows for generating ancestrally diverse reference lines (Ellis et al., 2025), reporting strategies tailored to minority populations (Zaaijer and Capes-Davis, 2021), and approaches to incorporating sex as a biological variable in MPS (Kouthouridis et al., 2022). Complementing these efforts, policy frameworks from the National Academies of Sciences, Engineering, and Medicine (NASEM) and the Global Alliance for Genomics and Health (GA4GH) now provide terminology and reporting standards for conceptualizing and documenting population diversity in genomic research (NASEM, 2023; Raven-Adams et al., 2024).

By strengthening infrastructures for inclusion and providing researchers with best practices to use and report population descriptors in genetics and genomics, these efforts have clarified and improved *how* diversity should be operationalized and documented in (biomedical) research. However, they do not address the prior question of *when* diversity should be incorporated, nor do they examine the methodological trade-offs involved in determining whether its inclusion is feasible or justified given the resource constraints that characterize many experimental settings. This latter question is particularly acute in hiPSC-based preclinical research, where high inter-donor genomic variability, technical noise, limited sample sizes, and the absence of standardized validation benchmarks complicate such decisions (Brunner et al., 2023; Volpato and Webber, 2020). Moreover, the frameworks developed by NASEM and GA4GH were specifically designed for genetics and genomics research practices that involve human participants. Hence, these frameworks do not always straightforwardly translate to hiPSC-based experimental systems where the empirical units are cultured cells.

To address this gap, this article presents a conceptual and practical framework for deciding when and how to integrate diversity into hiPSC-based research. We first examine the terminological, methodological, and normative challenges of defining and implementing diversity in (pre)clinical contexts. Building on the NASEM framework, we then provide recommendations for describing and reporting diversity in stem cell research. Next, we introduce a decision framework that specifies four criteria for diversity inclusion: (1) experimental purpose, clarifying what the study aims to explain or predict; (2) biological plausibility, assessing whether mechanistic, clinical, or epidemiological evidence supports a differential effect; (3) platform stability, evaluating whether the model is stable enough to distinguish experimental noise from true biological effect; and (4) statistical power, which ought to ensure the experiment can detect diversity effects reliably. We then demonstrate how the framework and recommendations operate in practice by applying them to three hiPSC case studies and consider what current experimental practices, research infrastructures, and institutional conditions in stem cell research imply for the use of the framework and for diversity inclusion more broadly. We conclude by reflecting on several of the framework’s limitations.

## Challenges in defining and operationalizing diversity in (pre)clinical research

“Diversity” generally refers to differences between individuals (e.g., race, ethnicity, sex, age, or disability) and their distribution within populations or model systems (Xu et al., 2020). Recent years have witnessed growing calls for greater diversity across biomedical research and its adjacent translational contexts. These calls reflect a broader recognition that inadequate diversity in biomedical research hampers the generation of reliable evidence about disease and undermines the equitable translation of research findings into clinical practice (Ghosh et al., 2022; Moore and Piddini, 2023). However, a central concern with such calls for more diversity is that they often fail to specify the meaning of diversity and related terms, which groups should be included, for what purposes, and under which conditions diversity effects can be reliably detected (Lee et al., 2022; NASEM, 2023). Without clarity on how diversity is defined and operationalized, population descriptors may be applied inconsistently, reducing comparability across studies and limiting what can be learned about when and how diversity matters in preclinical research.

Explicit analyses of diversity and its ethical, conceptual, and methodological implications are scarce in MPS and hiPSC research (Weidema et al., 2025). We therefore examine how diversity is defined and operationalized in genomics, animal models, and human cell-line studies, where researchers have confronted similar challenges in characterizing diversity. This allows identification of conceptual and methodological issues likely to arise in stem cell research and to clarify how constructs such as sex, gender, race, ethnicity, and ancestry can be more consistently defined and applied. We focus on two forms most relevant to preclinical contexts: (1) sex and gender, and (2) race, ethnicity, and ancestry. Other forms of diversity (e.g., education, occupation, geography, and disability) are also important to understand health disparities but are seldom integrated into preclinical models, primarily because biological correlates (e.g., DNA, biomarkers) are absent, less well defined, or context dependent.

### Diversity in sex and gender

Understanding how sex- and gender-related factors influence health is widely recognized as a fundamental challenge in biomedical research (Ritz and Greaves, 2022). While often treated as interchangeable in preclinical contexts, sex and gender are increasingly understood by both researchers and policymakers as distinct but interrelated categories that refer to different aspects of biology, behavior, and society (Box 1).

Box 1 **Sex and gender**

Sex refers to biological differences between males and females. In mammals, including humans, these differences are established at conception when an ovum fuses with a sperm that carries either an X or a Y chromosome, generally producing an embryo with characteristic XX or XY configurations (Mauvais-Jarvis et al., 2020). Other chromosomal patterns (e.g., XO, XXY, and XYY) also occur and represent biologically meaningful forms of sex-linked variation (Allegra et al., 2023). These differences form the genomic basis of sex and influence gene regulation and expression from the earliest stages of development. Importantly, the biological consequences of chromosomal sex extend beyond the presence of single genes, such as *SRY*, which typically resides on the Y chromosome and plays a key role in initiating testis development, and instead involve broader differences in gene content, expression, dosage compensation, and epigenetic regulation (Mauvais-Jarvis et al., 2020). These processes contribute to variations in hormonal profiles, reproductive anatomy, fat distribution, immune function, and growth. Although sex has historically been framed in binary terms as male or female, no single biological characteristic is either necessary or sufficient to define an individual’s sex, as every trait commonly associated with sex exhibits natural variation across species and members within those species (Ma et al., 2013; Ngun et al., 2011). Thus, instead of treating sex as a fixed binary, others propose that it is better understood as a constellation of sex-related traits, where no single trait determines membership of a specific category, and no trait is unique to one sex (Ritz and Greaves, 2022).

While the distinction between sex and gender has long been challenged (Butler, 1990), gender commonly refers to the roles, norms, and behaviors culturally associated with individuals presumed to belong to a particular sexual category. It also includes the norms and institutional structures that shape how individuals encounter and engage with their environments, including exposures, risks, access to resources, and self-understanding as gendered individuals (Heidari et al., 2016; Ritz and Greaves, 2022).

In animal and preclinical research, gender is often—though not by all (e.g., de Waal, 2022)—dismissed as irrelevant because it is seen as absent in animals. Nonetheless, scholars have examined how gendered assumptions about bodies and behaviors may influence the design and interpretation of research (Ankeny, 2023; Harding, 1986). For instance, Clough (2011) discusses how social norms (e.g., discouraging girls from getting dirty) might influence health outcomes in humans (e.g., allergy susceptibility) by shaping patterns of environmental exposure. This raises the possibility of using animal models to investigate whether similar exposures affect biological pathways relevant to those outcomes. Effectively, a socially mediated pattern in humans may motivate a research question that is pursued in animals via experimentally controlled conditions.

While the distinction is conceptually important, sex and gender overlap and often mutually shape how individuals experience and respond to health and disease in ways that complicate simplistic causal explanations of health outcomes (Ballantyne, 2023). For instance, men and women generally differ in bone homeostasis as a result of different hormone levels, which can be further compounded by (gendered) norms around physical activity or sun exposure, whether rooted in occupation, religion, or aesthetic convention (Fausto-Sterling, 2005). Similarly, engaging in competitive or nurturing behaviors can modulate testosterone levels (Gleason et al., 2009; Van Anders et al., 2015). In other words, the interplay between sex and gender complicates the assumption that observed biological differences are purely based on sex. As Ritz and Greaves (Ritz and Greaves, 2022) succinctly remark, “the routes linking cells and society are short, and the ways that social influences permeate the body and get under the skin are myriad” (2022)—that is, just as hormone levels influence behavior, behavior also influences hormone levels.

Most sex-related traits exhibit average differences between groups (e.g., hormone levels or size). In reality, however, there is substantial overlap between such categories, as some women may be taller than most men and some men may have hormone levels lower than those of most woman (Pape, 2021). Moreover, many sex-related traits (e.g., circulating hormone levels, immune function, and metabolic regulation) change over an individual’s life course and fluctuate across time scales ranging from hours to decades in both males and females (Hägg and Jylhävä, 2021; Koebele et al., 2022). Thus, treating male and female as homogeneous categories ignores this within-group variability and can lead to misleading overgeneralizations. For instance, the sedative Zolpidem was initially prescribed at lower doses for women based on average differences in drug clearance 8 hours after dosing. However, subsequent research showed no consistent sex-based differences in active drug concentration or functional impairment, raising concerns that some women were undertreated (Greenblatt et al., 2019).

A statistically significant average difference between sexes may suggest that sex- or gender-related factors are involved but does not imply that males and females function fundamentally differently or necessarily require separate forms of treatment (Pape, 2021; Ritz and Greaves, 2022). Comparing binary categories like male and female can be pragmatically useful, especially when mechanisms are unclear, but these categories should not be treated as explanatory endpoints. By doing so, researchers may stop short of asking more specific questions about the biological, social, or environmental processes that could be driving the observed variation (Greaves and Ritz, 2022).

### Diversity in race, ethnicity, and ancestry

Over the past years, health research has increasingly included considerations of race, ethnicity, and ancestry (Kanakamedala and Haga, 2012). Similar attention is emerging in genomics, stem cell biology, and cancer models, where the notion of ancestry is typically employed (Sirugo et al., 2019; Zaaijer and Capes-Davis, 2021). As discussed in what follows, diversity is socially framed in terms of race and ethnicity, while biological interpretations are associated with genomic ancestry.

#### Race and ethnicity

Race and ethnicity are among the most used classificatory categories in biomedical research and clinical practice. Race is typically used to group people based on perceived physical traits (e.g., hair texture, eye color, and skin tone) as well as presumed geographic origin (Root, 2017). In biomedicine, it shapes both population-level research, such as epidemiology and public health, and individual-level decision-making in clinical care (Lu et al., 2022; Root, 2017). Population-level approaches are committed to advancing the health of entire populations (Childress et al., 2002). This focus requires them to deal with aggregated health concerns and describe patterns in morbidity, mortality, and treatment response across large social groups. Statistical generalizations from such research are sometimes carried over into clinical care, where race is used to guide individual diagnosis or treatment (Root, 2017). This practice is contested because it assumes that average differences between racial groups are caused by biological factors associated with race. This leads to the use of race as if it reliably reflects biological differences, which may divert attention from more specific and potentially more relevant determinants of health, such as socioeconomic status, environmental exposures, or genomic variants. The use of race in this way has been described as a form of racial profiling that risks misdirecting clinical judgment and reinforcing biological essentialism (Khan et al., 2022; Lu et al., 2022).

Given the social and political weight of the term “race,” many researchers prefer to use “ethnicity,” which typically refers to shared cultural characteristics such as language, religion, dietary customs, and national or regional origins (Mensah et al., 2019). Ethnic categories are used to examine differences in disease risk and treatment outcomes linked to cultural or lifestyle factors and to guide culturally tailored healthcare (Anand et al., 2003). For example, Jewish populations are sometimes treated as an ethnic group when shared practices are considered relevant to health (Pearson and Geronimus, 2011).

Perhaps unsurprisingly, the term ethnicity is ambiguous and, like race, lacks a standard definition, may change over time, and often includes considerable heterogeneity within certain population groups (Lu et al., 2022). Furthermore, ethnicity involves identity markers that go beyond appearance. This can lead to interpretive ambiguity, as researchers and participants may apply the same label to culturally or racially distinct subgroups (Ford and Kelly, 2005). For instance, someone who identifies as or is considered “Hispanic” may differ significantly from others in the same group in terms of culture, nationality, or racial identity.

#### Classifying race and ethnicity

In biomedical research contexts, there are two primary methods for determining race and ethnicity: (1) self-report, where individuals identify their race by selecting from predefined categories, and (2) observer classification, where a researcher, clinician, or administrative staff member assigns a racial or ethnic label to the individual based on physical appearance and cues like name or accent (Lu et al., 2022; Root, 2017).

These approaches rely on the assumption that racial and ethnic identities are both identifiable and stable over time. However, research has indicated that self-reported race and ethnicity show notable variation across life stages and social contexts, particularly among individuals from multiracial or minority backgrounds (Liebler et al., 2017; Saperstein and Penner, 2012). For example, a recent analysis of US Bureau of Census data shows that approximately 6.1% of the respondents (9.8 million people) reported a different race and/or ethnicity in 2010 compared to 2000 (Liebler et al., 2017; Lu et al., 2022).

These shifts can reflect changes in self-understanding, social environment, or perceived social and material benefits associated with particular identities. For example, some individuals simplify their identification to a single race in adulthood, potentially influenced by significant life events such as marriage or leaving a childhood home (Perez and Hirschman, 2009). Others may adjust their responses based on the setting in which a question is asked: studies have shown that individuals sometimes report different racial identities at home than at school or work, depending on comfort or perceived safety (Liebler et al., 2017; Saperstein and Penner, 2012). The design of the research instrument itself (e.g., question-wording, available response options, and the mode of data collection) also influences how individuals report race and ethnicity (Perez and Hirschman, 2009; Saperstein and Penner, 2012). Together, these findings complicate the idea that race or ethnicity, once reported, function as stable or objective variables in biomedical research.

#### Ancestry

Whereas race and ethnicity refer to socially constructed categories, ancestry is typically used in genomic and preclinical research to denote an individual’s or group’s genetic (i.e., biological) origins (Khan et al., 2022; Mensah et al., 2019; Root, 2017). Ancestry can be defined in at least three ways: geographic ancestry, which refers to the general region or continent where an individual’s ancestors originated; genealogical ancestry, which is based on documented family lineages such as pedigrees or family trees; and genomic ancestry, which reflects patterns of inherited genomic markers an individual shares with certain ancestral populations (Lu et al., 2022; Mersha and Abebe, 2015).

In biomedical research, genomic ancestry takes central stage and is typically estimated by comparing segments of a person’s DNA to reference data from individuals with well-documented ancestral backgrounds (also known as Ancestry Informative Markers) (Mersha and Abebe, 2015). Although human genomes are more than 99.9% identical, the remaining 0.1% contains enough variation to reveal population-level differences (Hinds et al., 2005). Most of this variation consists of single-nucleotide polymorphisms (SNPs), which are small changes at specific positions in the DNA sequence (Mersha and Abebe, 2015). Some SNPs are more common in certain populations than others; in fact, around 86% of known variants are confined to single continental groups, with individuals of African descent showing the highest genetic diversity (Choudhury et al., 2020). These patterns reflect long-standing demographic and environmental histories, such as migration, isolation, and local adaptation.

Some ancestry-associated genetic variants have been found to influence molecular and cellular processes relevant to disease susceptibility and treatment response (Khan et al., 2022; Mersha and Abebe, 2015). These include differences in gene expression, protein function, and cellular behavior. For example, one study in Mexican Americans found that higher proportions of European ancestry were associated with more severe forms of asthma: for every 10% increase in European genomic ancestry, baseline FEV1, a standard measure of lung function, decreased by 1.7% (Salari et al., 2005), effectively illustrating how genomic ancestry can correlate with clinically meaningful variation in disease phenotypes.

### Recommendations for describing and reporting populations in stem cell research

Building on the conceptual distinctions introduced above, this section translates them into practical guidance for describing and reporting diversity in stem cell research. By doing so, we draw on the NASEM’s report *Using Population Descriptors in Genetics and Genomics Research* (2023), which articulates a broad set of recommendations and best practices for the use of race, ethnicity, ancestry, and related population descriptors in genomics. Table 1 reiterates a subset of the practices we believe bear directly on reporting population descriptors as general requirements for their selection, use, and documentation in stem cell research (Recs 1–8), and extends them with several stem cell-specific recommendations (Recs 9–12).

**Table 1:** Recommendations for describing and reporting populations in stem cell research

No.	Recommendation	Reporting guidance
1	Select population descriptors in relation to the scientific question.	There is no default or universally appropriate population descriptor, so descriptor choice must be justified by the study’s purpose and intended inference, not by convention or data availability.
2	Do not use race as a biological proxy.	Race should not be used to represent genomic variation or genomic ancestry, nor should racially defined groups be retrospectively interpreted as biologically meaningful categories.
3	Define population descriptors explicitly.	Researchers must state what each descriptor is intended to capture and how it is conceptualized to avoid ambiguity between race, ethnicity, ancestry, geography, or nationality.
4	Attend to gene *x* environment interactions.	Where environmental or contextual factors are relevant, they should be evaluated directly. Population descriptors should not be treated as substitutes for such factors, but when they are nonetheless used as proxies, their role, relevance, and limitations must be stated explicitly.
5	Document how labels are assigned.	Studies should report how population labels were obtained, including whether they are self-reported, assigned, inferred from data, or derived from existing records.
6	Apply descriptors consistently across all samples.	If a descriptor is used, it should be applied uniformly to all individuals or samples. Mixing different descriptor types within a study undermines the study’s interpretability.
7	Avoid typological population thinking.	Population descriptors should not imply discrete, homogeneous, stable, or hierarchical biological groups. Researchers should therefore specify how ancestry is operationalized and report within-group variation by using, for instance, continuous measures such as genetic distance metrics (Ding et al., 2023).
8	Justify grouping decisions.	Any grouping of individuals or samples must be explained, including the rationale for grouping and how it relates to the study’s aims, particularly when working with legacy data.
9	Specify operative donor characteristics and implications for the study’s purpose.	When population descriptors are used, researchers should identify which donor-linked biological features are expected to remain operative in the stem cell model after reprogramming and differentiation and clarify how this might constrain or complement a study’s goal.
10	Report incomplete donor metadata.	When donor characteristics or contextual variables are unavailable, researchers should report this absence and clarify how this might influence interpreting potential diversity effects in the stem cell model.
11	Define sex relative to the research aim.	When sex is included as a variable, researchers should specify which biological feature it is intended to represent (e.g., chromosomal or hormonal) and why this specific feature is appropriate for the level of analysis and research question under investigation (Richardson, 2022).
12	Avoid treating binary sex categories as explanatory endpoints.	When sex-linked effects are hypothesized, researchers should measure the underlying biological variation at an appropriate resolution rather than relying solely on binary sex categories, for example by using quantitative or transcriptomic measures of sex-linked gene expression (Yang and Rubin, 2023).

Recommendations 1–8 are adapted from NASEM’s (2023) reporting guidelines for genomics research. Recommendations 9–12 are additions that address challenges specific to stem cell research.

Applying NASEM’s genomics-oriented reporting practices to stem cell research raises several distinct methodological and conceptual questions. A central issue is that donor-linked biological states are not uniformly preserved during reprogramming and differentiation, which might complicate assumptions about what a specific population descriptor can meaningfully represent *in vitro*. Female hiPSC lines, for example, often exhibit altered, unstable, or heterogeneous patterns of X chromosome inactivation, such that “female” hiPSC-derived cells do not consistently correspond to the regulatory states found in somatic tissues (Raposo et al., 2025). Furthermore, sex- and ancestry-associated effects often depend on interactions between biology and environment (i.e., lived contexts). Because these contexts are largely stripped away under standardized *in vitro* culture conditions, such effects may not manifest, or may manifest differently, in stem cell systems (Bisogno et al., 2020; Schaniel et al., 2021).

Under these conditions, the meaning and use of population descriptors in stem cell models cannot be assumed to mirror their meaning and use in genomic, clinical, or epidemiological models, and treating them as interchangeable across these and other domains risks overstating what they can represent about donor background and, by extension, what inferences they can support. Therefore, items 9, 11, and 12 specify several additional recommendations that address how population descriptors should be selected, interpreted, and reported in light of these constraints, while item 10 addresses the related but distinct issue of how absent or incomplete donor metadata should be documented.

Taken together, these practices and/or recommendations establish reporting standards for diversity in stem cell-based preclinical research that, when properly adhered to, can improve clarity, comparability, and reliability across studies. Table 2 supports this aim by providing a set of working definitions of key diversity-related terms as we propose they should be operationalized in stem cell research systems. However, neither these recommendations nor the working definitions determine when and how the inclusion of diversity variables is methodologically warranted across hiPSC research contexts; this issue is addressed in the following section.

**Table 2:** Working definitions of diversity in preclinical stem cell models

Term	Working definition	Notes on relevance and limitations
Sex	Refers to chromosomal, hormonal, transcriptional, and anatomical characteristics that together contribute to biological dimorphism.	In cell lines, sex differences are present at the level of sex chromosome composition and associated gene expression, but not at the level of hormones or whole-body traits (Pottmeier et al., 2024). Transcriptomic sex indices (Yang and Rubin, 2023) allow sex to be represented as a continuum rather than a binary category.
Gender	Socially constructed roles, norms, and behaviors that influence health, disease, and research participation.	Gender does not directly manifest in cell lines but affects research indirectly through differential stress, nutrition, or healthcare access that leave epigenetic or hormonal marks on donor tissues and through structural biases that determine which samples and diseases enter research.
Race	Socially constructed classification based on perceived physical traits or presumed geographic origin; not a biological trait in cell lines.	Although race is not a biological variable in stem cell models, it is often recorded in donor metadata or used in recruitment criteria for cell line panels (Chen et al., 2021). This can lead to conflation of social and biological categories, introducing biases or overgeneralizations. Race-related metadata in cell line studies must be interpreted with caution to avoid falsely equating socially defined race categories with underlying genomic or cellular differences.
Ethnicity	Socially defined group identity based on shared culture, language, religion, or national origin; not a biological trait in cell lines.	Like race, ethnicity appears in donor metadata but does not correspond directly to genomic ancestry. Its meaning varies across contexts, and ethnicity-based comparisons may obscure within-group heterogeneity or reinforce social stereotypes
Ancestry	Genomic variation reflecting inherited DNA markers shared with specific ancestral populations; typically inferred from genomic data (e.g., SNPs, haplotypes).	Ancestry is biologically relevant for cell models because it captures genomic differences that can influence cellular phenotypes or drug response. It should be quantified using explicit inference methods or continuous metrics such as genetic distance (Ding et al., 2023) rather than categorical continental labels.
Interaction effects	Combined influences among genomic, environmental, sex, or other trait-based variables.	These interactions can shape phenotypic outcomes in ways that single-factor analyses miss. “Village-in-a-dish” designs that co-culture hiPSCs from multiple donors under shared conditions enable controlled analysis of genotype *x* environment and genotype *x* sex effects.

## Determining when and how diversity matters in stem cell research

To determine when and how diversity should be incorporated into stem cell-based preclinical research, this section examines the conditions under which diversity variables can meaningfully inform experimental design. In doing so, we first outline key challenges that shape diversity inclusion in hiPSC-based studies and then develop a threshold framework to evaluate when and how inclusion is warranted in such studies.

### Stem cells and some challenges for inclusion

To determine when and how diversity matters in stem cell research, this section examines four challenges that complicate decisions about inclusion: experimental purpose, limited knowledge of relevant mechanisms, platform heterogeneity, and low statistical power.

First, stem cell research encompasses a wide variety of experimental systems, cell types, platforms, and research aims. In such a heterogeneous research landscape, practices of inclusion cannot plausibly function as a singular factor with a uniform role across studies. Whether diversity is relevant, peripheral, or intentionally excluded depends on what the study is trying to establish in that context. Thus, consistent with the guidance discussed above, we argue that the role of diversity should be assessed in relation to a model’s *experimental purpose*—or, more precisely, to the purposes and values of the researchers who use the model (Fagan, 2021).

The second challenge concerns limited knowledge of the mechanisms through which variables such as sex, ancestry, or genotype may affect phenotypes in early-stage hiPSC-based preclinical research. In the absence of such knowledge, researchers necessarily rely on extrapolation—that is, on treating results obtained in one distinct biological or experimental context (e.g., epidemiological populations, animal models, or clinical settings) as evidence for making inferences about another (Baetu, 2016; Steel, 2007). While observations from these contexts may provide indications of how sex, ancestry, or genotype would influence experimental readouts, it is often unclear whether the same mechanisms can operate, or operate in the same way, within particular hiPSC-derived models.

A third, overlapping source of uncertainty is the biological and technical variation present in hiPSC lines and platforms. Donor background, reprogramming history, somatic mutations, epigenetic state, passage number, and culture conditions all shape stem cells’ pluripotency, differentiation capacity, and gene expression (Fossati et al., 2016; Volpato and Webber, 2020). This variability is further compounded by the disunified nature of stem cell research (Fagan, 2021), in which laboratories implement hiPSC systems through locally developed practices and experimental protocols. Consequently, differences observed between donors or groups may reflect platform-specific or technical variation rather than diversity-related biological effects, limiting the reliability and transferability of such findings across experimental contexts. Although statistical modeling can estimate and adjust for particular sources of variation, this requires clear and stable categorization, which may be difficult to achieve for variables such as sex (see Section diversity in sex and gender) and, most pertinently, ancestry (see Section diversity in race, ethnicity, and ancestry).

Finally, hiPSC studies are currently often underpowered because many study designs do not accurately account for genomic diversity, e.g., by using only a very limited number of independent donor lines per treatment or condition (Brunner et al., 2023). Integrating diversity into studies that are underpowered or lack the design features required to capture relevant variation risks producing unreliable results, including false negatives, false positives, and exaggerated effect size estimates (Button et al., 2013). Although results from underpowered or poorly specified studies are unreliable, they may still be cited in subsequent research or inform preclinical decisions, thereby propagating flawed evidence and misleading further investigation.

Combined, differences in experimental purpose, limited mechanistic knowledge, platform heterogeneity, and low statistical power define four criteria that, we argue, constrain when and how diversity variables can be meaningfully included in hiPSC-based studies. On this basis, we propose the following guiding questions for determining when diversity can be included in a reliable and methodologically justified way.

1. **Are the experimental purpose and research question clearly defined in relation to the model system’s context?** hiPSC models are used for different purposes at different stages of research, ranging from early exploratory or proof-of-concept studies to late-stage preclinical applications approaching first-in-human trials. These purposes shape what kind of claims a study is meant to support. For example, early exploratory studies often aim to establish whether a phenotype can be induced or a protocol works at all, whereas more advanced comparative studies assess differences across lines or groups, and late-stage applications are typically designed to support robust comparisons and translational inferences at the population or sub-population level.
2. **Is there a plausible mechanistic basis to expect diversity-related effects in the hiPSC under study, and what are the implications of this for how diversity should be included?** This question is intended to clarify how diversity-related differences could plausibly arise within the experimental system and how this bears on the design, resourcing, and interpretation of diversity-oriented analyses. Situations in which mechanistic plausibility may be limited or contested include cases where evidence from other model systems does not clearly translate to hiPSC-based models, where published findings are inconsistent without clear methodological explanations, where clinical or epidemiological associations lack established mechanisms, or where preliminary pilot data suggest possible effects but remain underpowered.
3. **Is the model platform sufficiently stable and sensitive enough to detect relevant effects?** Observed differences between cell lines may reflect biological variation, technical variation, or their interaction. Benchmarks for readiness will differ by model system, cell type, and endpoint but should follow field standards such as the International Society for Stem Cell Research’s *Standards for Human Stem Cell Use in Research* (ISSCR, 2023) and the Economic Co-operation and Development’s *Good In Vitro Method Practices* (Economic Co-operation and Development, 2018).
4. **Is the study adequately powered to support reliable inferences?** Power requirements depend on effect size, sample design, and the degree of dependency among observations from the same donor line (Brunner et al., 2023). Dependency, or nesting, in the data indicates that datapoints from one cluster, e.g., those derived from one particular hiPSC line, exhibit greater similarity to each other than to datapoints from another hiPSC line. Thus, when including hiPSC lines from a heterogeneous donor pool to recapitulate diversity effects, a higher degree of dependency in the resulting dataset is anticipated, which needs to be quantified and accounted for in subsequent statistical analyses (Aarts et al., 2014). Higher dependency negatively affects attainable power, meaning that a larger number of independent hiPSC lines is necessary to achieve sufficient power (Brunner et al., 2023; Button et al., 2013).

Applying the framework to concrete experimental settings will of course reveal situations in which only some of the criteria can be met. The inability of an experiment to satisfy all of the framework’s conditions, however, should not be seen as a motivation for researchers to engage in practices of definite exclusion. Rather, failure to meet all criteria identifies the specific limitations that prevent meaningful inference at a *particular moment of model development* and at the same time indicates which elements of the experimental setup would need to be adjusted for inclusion to become both reliable and feasible, such as increasing the number of independent cell lines to improve statistical power.

## Applying the framework in experimental design

**Figure 1:**
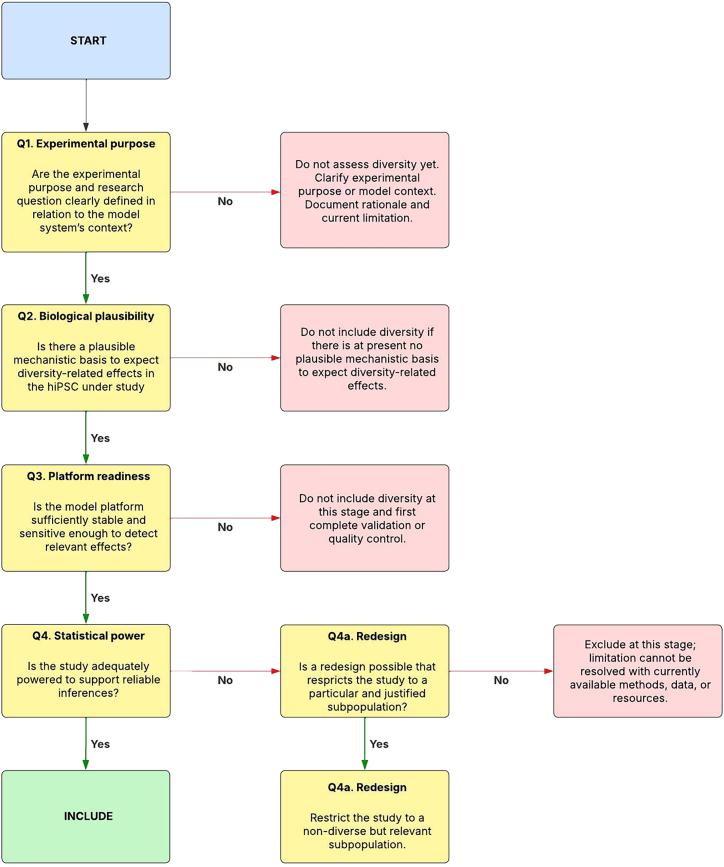
Decision flowchart for assessing when to proceed with diversity inclusion in hiPSC-based research This stoplight decision flowchart guides decisions about whether to proceed with the inclusion of diversity variables in hiPSC-based preclinical research. The flowchart operationalizes four questions about experimental purpose, biological plausibility, platform readiness, and statistical power, respectively. A “Yes” at each step indicates that the corresponding condition is sufficiently met and that it is appropriate to proceed to the next one. A “No” indicates that the condition is not met and that diversity should not be included at that stage of model development. When inclusion is blocked due to insufficient statistical power despite a clear experimental purpose, biological plausibility for diversity-related effects, and a stable platform, the flowchart indicates when a deliberate redesign that restricts the study to a justified subpopulation may be considered as a responsible alternative to premature comparative analysis.

Stem cell models are used in three primary fields of preclinical research: studying human physiology, disease modeling, and (pharmaco)safety or toxicity testing (Rowe and Daley, 2019; Shi et al., 2017). To illustrate how the framework operates in practice, we apply it to three hiPSC-based platforms, each representing one of these different fields of enquiry (Box 2). In each case, the framework is applied sequentially by addressing the four questions developed in the previous section. A positive answer to question 1 is required to proceed to question 2, and so on. If a question is answered negatively, inclusion is not methodologically warranted at that particular stage of model development. The case analyses then outline what changes to the experimental system would be required for inclusion to become analytically meaningful. Throughout this process, the recommendations in Table 1 provide additional structure by guiding how population descriptors should be selected and documented when diversity-related variables are introduced, expanded, or deliberately deferred. The decision logic of the framework is shown in Figure 1.

Box 2 **Examples of stem-cell-based preclinical research**

**Example 1: Skeletal muscle development**: skeletal muscle develops through a sequence of progenitor stages that give rise to organized and contractile fibers. Chal et al. (2015) reconstructed these developmental steps *in vitro* by directing hiPSCs through presomitic mesoderm and early myogenic states. The resulting cultures formed elongated, multinucleated fibers with organized sarcomeres and spontaneous contractions. In longer-term cultures, a small population of Pax7-positive cells emerged that resembled satellite-cell precursors described *in vivo*. Satellite cells are the stem cells that sit alongside muscle fibers in the body and support muscle repair after injury, so the presence of Pax7-positive cells in this system suggests that aspects of this regenerative capacity also emerge *in vitro*. To assess whether these cells exhibited satellite-cell properties, the authors transplanted them into dystrophic mouse muscle, where they formed dystrophin-positive fibers and contributed Pax7-positive cells beneath the basal lamina, demonstrating regenerative capacity *in vivo* (Chal et al., 2015, 2016).

**Example 2: Cardiac disease modeling**: Barth syndrome (BTHS) is a rare X-linked mitochondrial heart disease caused by mutations in the *tafazzin* (*TAZ*) gene that disrupt normal lipid processing and lead to muscle weakness and progressive heart failure in affected males (Clarke et al., 2013). By generating hiPSC-derived cardiomyocytes (hiPSC-CMs) from two unrelated BTHS patients (BTH-H and BTH-C), Wang et al. (2014) modeled the disease on a heart-on-chip system capable of measuring contractile force. Across both lines, the CMs exhibited consistent abnormalities, including weak contraction, disrupted sarcomere organization, and pronounced mitochondrial dysfunction, including elevated levels of reactive oxygen species (ROS). Although cellular adenosine triphosphate (ATP), the molecular unit responsible for intracellular energy transport, could be restored by shifting metabolism toward glycolysis, the contractile defects persisted. Together, these findings indicate that impaired contraction arises from mitochondrial dysfunction, leading to excessive ROS production rather than from a primary loss of ATP.

**Example 3: Hepatocyte-based toxicity testing**: alpha-1 antitrypsin (*AAT*) deficiency is an inherited liver disorder in which a mutation of the *AAT* protein accumulates within hepatocytes and can lead to progressive liver damage. Choi et al. (2013) used hiPSC-derived hepatocytes from *AAT*-deficient patients in a high-throughput screening platform to test 3,131 clinically approved compounds for their ability to reduce intracellular *AAT* accumulation. An initial screen was performed in one patient-derived line, after which candidate compounds were tested across four independent hiPSC lines (iAAT2, iAAT3, iAAT25, and iAAT45). Five compounds showed reproducible reductions in intracellular *AAT* accumulation across lines, including carbamazepine, a drug previously shown to promote clearance of misfolded proteins in animal models of the disease.

### Skeletal muscle development and the identity of Pax-7-positive cells

In the skeletal muscle case, the experiment’s primary goal is to establish whether the developmental and regenerative features of human muscle can be reproduced and functionally validated *in vitro* (Q1). Chal et al. (2016, 2015) developed hiPSC-based models that accurately reproduce key aspects of myogenic differentiation and tissue organization, including a Pax7-positive cell population that resembles satellite cell precursors.

Sex differences in skeletal muscle physiology are well documented *in vivo*: female muscle generally contains more type I and IIA fibers supportive of endurance, while male muscle has more type IIX fibers associated with greater strength (Haizlip et al., 2015). These are structural and metabolic differences that could make it plausible for donor sex to influence experimental outcomes (Q2).

However, the model has not yet demonstrated autonomous muscle regeneration *in vitro*; regenerative capacity is inferred from transplantation into dystrophic mouse muscle rather than shown within the culture system itself. Until the study’s primary claim is established within the *in vitro* system, any attempt to compare donor lines would test differences in an unvalidated function rather than variation in a stabilized regenerative capacity (Q3).

In this case, inclusion of diversity variables would be premature, and progress toward their inclusion depends first on stabilizing the biological target itself. This requires demonstrating regeneration *in vitro*, for example through reproducible injury-and-repair assays that show autonomous activation, contribution, and maintenance of the Pax7-positive compartment. Establishing such a platform-internal regenerative phenotype would then clarify what counts as successful and reduce ambiguity on cell identity and function. Once this function is established, it becomes appropriate to expand the number of independently derived donor lines and examine whether regenerative capacity varies across backgrounds. Until then, it is essential to adequately document donor provenance and characterization (Recs. 5, 9) while avoiding premature grouping or comparison across backgrounds (Rec. 8).

### Barth syndrome, cardiac disease modeling, and limited cell lines

In the cardiac case, the experimental aim was to identify the cellular mechanisms through which mutations in the *TAZ* gene cause contractile dysfunction in Barth syndrome (Q1). Using two patient-specific hiPSC-derived cardiomyocyte lines in a heart-on-chip platform, Wang et al. (2014) showed that contractile defects arise from mitochondrial dysfunction and excessive ROS rather than ATP depletion.

Although Barth syndrome is X-linked, considerable clinical heterogeneity has been observed among affected individuals, including variation in the presence and severity of cardiomyopathy, neutropenia, skeletal myopathy, and age of onset (Clarke et al., 2013). This heterogeneity plausibly reflects differences in how the *TAZ* mutation is expressed across tissues and developmental stages, making it reasonable to expect that patient-specific biological differences will affect cellular phenotypes and hiPSC-based experimental readouts (Q2).

The consistent reproduction of contractile and mitochondrial abnormalities across both patient-derived lines, as well as the platform’s ability to distinguish between restored ATP levels and persistent contractile dysfunction, indicate that the model is sufficiently stable and sensitive to detect relevant diversity effects (Q3). However, the inclusion of only two patient-derived lines means that the study cannot support generalizable inferences beyond a shared disease mechanism and should not be taken to characterize patient-to-patient differences in Barth syndrome (Q4).

Making patient-to-patient variation tractable in this setting requires expanding the number of independently derived Barth syndrome lines so that differences in cellular phenotypes can be examined directly (Rec. 8). Pairing patient lines with isogenic corrected controls would further help separate effects attributable to the *TAZ* mutation from modifiers associated with individual genomic backgrounds, thereby clarifying what sources of variation the model is intended to capture (Rec. 1). Under this design, documenting donor background characteristics is essential to interpret differences across lines and specify what kinds of variation the model is intended to capture (Recs. 5 and 9).

### Hepatocyte toxicity screening

In the hepatocyte case, the experiment’s aim was to identify drug candidates capable of reducing the accumulation of mutant AAT in liver cells (Q1). To this end, Choi et al. (2013) utilized hiPSC-derived hepatocytes from patients with AAT deficiency in a high-throughput screening design, initially testing more than 3,000 compounds and then evaluating a smaller set of promising candidates across four independent hiPSC lines.

There is direct evidence that biological sex is associated with systematic differences in the clinical expression of *AAT* (Q2). Registry data show that among *PiZZ* individuals (one of the major *AAT* genotypic classes), men have a higher prevalence of chronic obstructive pulmonary disease (COPD) and liver disease, whereas women show higher rates of bronchiectasis as well as higher symptom burden and exacerbation risk, even after adjustment for smoking history and other covariates (Ersöz et al., 2025).

The model platform appears sufficiently stable and sensitive for its intended purpose (Q3). By evaluating candidate compounds across multiple independent hiPSC lines and retaining only those with consistent effects, the design allows reproducible and disease-relevant reductions in mutant *AAT* accumulation to be distinguished from line-specific responses. However, with only four patient-derived hiPSC lines, the study is not designed to support strong population-level generalizations or to assess inter-individual variability in drug response (Q4). Within these limits, the design deliberately filters for effects that are reproducible across the included donor lines, effectively prioritizing the assessment of whether the compound is at all robust without claiming population-wide applicability.

Making diversity analytically relevant in this setting would require a different use of inter-line variation within the screening design. Rather than treating differences across hiPSC lines solely as a criterion for discarding compounds, the study would need to retain and analyze differential drug responses as outcomes of interest. This should be done alongside the explicit documentation of donor background variables (Recs. 1, 5, 9) and would require justification of the strategies through which donors are grouped and compared (Rec. 8).

### Synthesis

Two general insights emerge from applying the framework to the three cases. First, it shows that decisions about inclusion are essentially judgments about the *epistemic capacity* of hiPSC-based preclinical models. By epistemic capacity, we mean the situated ability of an experimental system to generate outputs that can legitimately count as evidence for a specified class of claims, which in our case are about diversity-related effects (cf. Leonelli, 2016). Indeed, researchers can sincerely and explicitly decide that diversity matters for what they want to study, but that decision does not by itself determine how and when diversity can be meaningfully incorporated. Instead, such decisions should be guided by whether the experimental system is organized in a way that could, in principle, support the meaningful analysis of diversity-related effects. This involves taking conscious note of how the experimental system is organized for inquiry, including the preparatory choices that constitute its material setup (e.g., cell line selection, differentiation protocols, and measurement instruments), the comparative structure of the experiment (i.e., how differences are defined, stabilized, and assessed), and the system’s stage of development (e.g., whether it is configured for exploratory investigation or for mature screening applications).

An important consequence of this way of framing hiPSC-derived evidence is that questions about diversity (and other targets) cannot be addressed independently of the experimental systems in which they arise. In practice, evidence relevant to such questions does not pre-exist “out there” in donor populations, passively waiting to be uncovered simply by applying a model. Rather, evidence is *generated* through experimental work in multiple and non-equivalent ways (Russo, 2022), depending on how hiPSC-based systems are materially and methodologically arranged. Differences in cell line selection, differentiation and maturation protocols, culture conditions, readout technologies, and even conceptual framing contribute to how biological phenomena are operationalized, compared, and interpreted and thus to which biological differences can be stably identified and assessed as evidence. From this perspective, questions about diversity should not be approached in absolute terms but instead assessed in relation to the experimental practices through which hiPSC-based systems make variation available as evidence (Ankeny and Leonelli, 2021; Leonelli, 2016; Russo, 2022).

Second, applying the framework to the cases also shows how it can be used to identify the conditions under which diversity-related claims can be meaningfully supported across different experimental contexts. More specifically, instead of asking in general whether diversity is or should be included, the framework (re)directs attention to the specific features of an experimental configuration that determine which diversity-related questions can reasonably be asked and when diversity-related inferences can be reliably drawn. In the skeletal muscle case, diversity-related questions remain premature because the biological target itself is still being stabilized within the system. In the cardiac case, the disease mechanism can be reliably modeled, but diversity-related inference requires enough independent lines to support comparisons across individuals. In the hepatocyte case, multiple lines are already in use, but diversity-related inference warrants a different framing of the research question that explicitly treats diversity as an object of enquiry.

Importantly, under such constraints, the response should *not* be to proceed with the comparison anyway, but rather to make an explicit and justified choice about how diversity should be handled at that stage of model development. This may involve deferring comparative analysis for the time being or, in some cases, deliberately focusing on a particular and relevant subpopulation, for example by studying cells from one sex when a disease shows well-established sex differences in incidence or clinical presentation. While such approaches do not measure diversity effects directly, they offer a more responsible alternative to treating donor background as irrelevant or selecting available hiPSC lines without justification.

By making explicit how different experimental systems support different kinds of diversity-related claims and inferences, the framework allows diversity-related questions to be properly evaluated relative to the concrete aims, capacities, resources, and limitations of particular platforms instead of against a single and abstract standard. Specifically, it brings into view that what counts as a reasonable moment and manner to address diversity depends both on what a model is built to do and on how far its development has progressed. We can thus see how the framework is not meant to function as a straightforward test that researchers can simply pass or fail and instead serves to clarify when and how diversity-related inquiry becomes epistemically tractable in stem cell research.

## Discussion

In this paper, we outlined recommendations for describing and reporting diversity in stem cell research and developed a four-criterion threshold framework for assessing when diversity-related inference is scientifically justified, which we then applied to three hiPSC case studies. Rather than prescribing general rules for inclusion, the framework is intended as a diagnostic tool that helps to identify any potential limitations and what should change for diversity-related analyses to be carried out reliably. In what follows, we reflect on several broader implications of this approach, outline a few remaining tensions, and provide several directions for future research.

First, our framework highlights a basic feature of how early preclinical and experimental platforms are typically developed. Early studies are usually designed with a strong emphasis on control, often by working in highly simplified and standardized experimental environments. The most fundamental aim of these experimental environments is to reduce variability in order to establish stable, reproducible, and comparable experimental systems (Ankeny et al., 2023; Voelkl et al., 2018; Würbel, 2000). In hiPSC-based preclinical research, such a “control-first” approach often involves working with a limited number of donor lines, which in practice tend to come from populations that are already well represented and characterized in existing repositories. Underrepresented groups, whose donor material is less commonly available at this stage, are therefore less likely to be included in early platform development. The biological variation typically associated with these groups will thus play a smaller role in shaping what the system treats as a normal baseline, which sources of variation are considered negligible, and which effects are detectable. In other words, what presents as a stable and generalizable platform may in fact reflect the biological profile of an already well-characterized subset, such that if a more diverse set of donor lines were to introduce relevant differences in baseline characteristics or sources of variation, this could, theoretically, indicate the need for further iteration in platform development.

While our framework does not in itself resolve this equity problem, it does provide practical guidance on how to handle it responsibly by requiring explicit assessment of what the experimental system can and cannot support. A particular consequence of leaving such assessments implicit is that it often remains unclear why diversity is absent from a study. Indeed, it may be irrelevant to the research question, unavailable in the data, or unsupported by the experimental system, but when these reasons are not documented, the downstream consequences of early design choices remain obscured, making it harder for later researchers to see which features of the system would need to change to better support diversity-related analysis. The reporting recommendations outlined in tandem with the framework provide an additional means to further address this issue: by guiding how decisions to exclude diversity should be documented and justified and by encouraging researchers to specify what information is missing and why certain variables are *not* analyzed, the recommendations help keep such limitations visible and make it easier for subsequent studies to identify where platforms should be extended or modified to better support diversity-related analysis.

Second, when laboratories are willing to pursue diversity-oriented questions at an exploratory level, constraints on sample size, donor access, and resources often leave hiPSC studies underpowered (Brunner et al., 2023). These limitations seem structural rather than methodological, which raises the question of whether the field can build power cumulatively (and collectively) rather than expecting each lab to achieve it independently.

Here, the challenge is not simply whether diversity-related analyses are appropriate but more so what conditions are required for sufficient power to be achieved in practice. Funding structures could play an important role in this context, both in enabling larger and more diverse study designs, as well as in shaping expectations about when and how diversity must be included in experimental work. But without sustained efforts to expand representation across hiPSC repositories and to improve the collection and documentation of donor characteristics, adequately powered diversity analyses will remain constrained, regardless of individual laboratories’ intentions. Within these constraints, however, the framework should not be read as dismissive of small-scale studies or as a statement in favor of performing excessively large studies in all cases. Small studies may still be conducted, but their findings need to be interpreted and presented in light of the effect size boundaries they can reliably detect: for very large effect sizes (i.e., very large differences between study groups), small sample sizes may suffice, while smaller effect sizes (i.e., more subtle differences between study groups) typically require larger sample sizes. Thus, rather than treating statistical power as a fixed threshold that is either met or not, an *a priori* definition of the range of effect sizes considered relevant to the research question can inform appropriate study design (Ashton, 2013; Ioannidis, 2005).

These issues illustrate that producing reliable diversity knowledge in hiPSC research cannot be achieved through improvements in individual studies alone. Although the framework diagnoses readiness for inclusion at the level of single experiments, that readiness is also shaped by broader organizational, infrastructural, and institutional conditions. This motivates several directions for further research.

First, the decentralized character of stem cell research also means that (diversity-related) knowledge is primarily produced locally within individual laboratories. Whether a reported diversity effect comes to count as meaningful and translatable depends on how results circulate, are taken up, and are coordinated across research groups. This makes the social organization of stem cell research itself an important object of further study (Fagan, 2021). Work in philosophy of science in practice, science and technology studies, and the history and philosophy of science offers tools, such as Ankeny’s and Leonelli’s notion of “repertoire” (Ankeny and Leonelli, 2016), to map how techniques, standards, materials, skills, and norms are aligned in different parts of the field and how these alignments shape what kinds of diversity knowledge can be produced and shared.

The reporting recommendations developed in this paper can be understood as a step in the direction of promoting greater clarity and consistency in how diversity variables are described and reported at the level of individual studies. However, the ways in which such locally produced knowledge is subsequently coordinated, compared, and stabilized across laboratories remains an open question and an important area for future empirical and conceptual research.

Second, stem cell models are commonly evaluated in terms of how well they “recapitulate” human biology and how tightly experimental conditions are “controlled.” Yet, what counts as adequate recapitulation and control is rarely made explicit and can differ across experimental contexts. As discussed above, diversity is typically introduced only once a model is judged to be sufficiently controlled, but this judgment is context-specific and depends on how biological and technical variations are interpreted within a given system. Because stem cell models differ in how biological and technical variations are handled in practice, and because the criteria by which variation is judged acceptable are often left implicit, further conceptual work is needed to clarify when a model is considered “controlled enough” for inclusion to become feasible and how this judgment structures diversity research in practice.

Third, the diversity of collections held in stem cell repositories and biobanks remains a central condition for enabling diversity-related research in practice. Efforts to expand such diversity have been unfolding over the years and include trust-building, sustained community engagement, transparent governance of long-term storage and reuse, and the inclusion of community-relevant expertise in research design (Claw et al., 2018; Lee et al., 2019; Mensah et al., 2018). However, recent systematic evaluations of stem cell research infrastructures suggest that diversity in collections alone is insufficient if it is not accompanied by infrastructures that support the reliable documentation, identification, and reuse of donor-related information. In particular, limited adoption of persistent identifiers, uneven metadata standards, and restricted interoperability across registries and biobanks can undermine the visibility and reuse of diverse cell lines once they are generated (Hu et al., 2025). Given their joint role in shaping which diversity-related questions can be reliably addressed over time, we argue that continued attention to both the composition of stem cell collections and the infrastructural conditions under which they are registered, described, and shared remains an important avenue for further research.

## Limitations

Like any conceptual framework, the one proposed here has several limitations, too. First, it does not provide fixed thresholds or quantitative rules for determining when diversity should be included but instead relies on contextual judgment and transparent justification. While this flexibility is intentional, it also means that different researchers may arrive at different conclusions when applying the framework to similar experimental settings. Second, the framework is designed to support decisions within existing experimental constraints and therefore cannot resolve broader structural limitations of hiPSC research, such as limited availability of diverse donor lines or unequal access to resources. Third, the framework focuses on methodological justification rather than on downstream ethical, clinical, or policy outcomes and thus does not by itself guarantee equitable translation of findings. Finally, although the framework is grounded in current practices and case studies, its applicability will need to be reassessed as stem cell platforms, analytical methods, and evidence standards continue to evolve.

## Conclusion

Based on an examination of how diversity is defined and operationalized across biomedical research and an analysis of how population descriptors are used in genomics and translated to cell-based models, this paper presents a framework to guide decisions about when and how diversity should be incorporated into stem-cell-based preclinical research. The framework links inclusion decisions to four criteria (experimental purpose, biological plausibility, platform readiness, and statistical power) that together clarify what kinds of diversity-related questions a given experimental system can reasonably support. Rather than prescribing uniform rules for inclusion, it offers a practical way to assess when and how diversity-related analyses are appropriate in a particular experimental context and complements existing reporting recommendations for describing and reporting populations in stem cell research. Taken together, this work aims to support clearer and more transparent decision-making in iPSC research, while helping to balance diversity-related considerations with other priorities in preclinical research, such as reproducibility and standardization, thereby improving the clarity, comparability, and downstream effects of stem-cell-based preclinical research.

## Data and code availability

All data analyzed during this study were publicly available at the time of submission.

## Acknowledgments

We would like to sincerely thank two anonymous reviewers for their careful and substantive engagement with this paper. Their comments genuinely led to significant improvements, and we are grateful for the time and thought they invested in this work.

Authors receive(d) funding through reNEW, the Novo Nordisk Foundation Center for Stem Cell Medicine (NNF21CC0073729). J.W., C.M., and H.L. receive funding through the 10.13039/501100003246Dutch Research Council (NWO; project no. 184.036.006).

## Author contributions

Conceptualization, J.W. and N.d.G.; methodology, J.W.; investigation, J.W.; data curation, J.W.; writing original draft, J.W.; writing review & editing, J.W., N.d.G., C.M., H.L., M.M., and M.d.V.; supervision, N.d.G., M.d.V., and C.M.; funding acquisition, M.d.V. and C.M.

## Declaration of interests

The authors declare no competing interests.
